# [Retracted] Differential effects of PXD101 (belinostat) on androgen-dependent and androgen-independent prostate cancer models

**DOI:** 10.3892/ijo.2025.5717

**Published:** 2025-01-03

**Authors:** Giovanni Luca Gravina, Francesco Marampon, Ilaria Giusti, Eleonora Carosa, Stefania Di Sante, Enrico Ricevuto, Vincenza Dolo, Vincenzo Tombolini, Emmanuele A. Jannini, Claudio Festuccia

Int J Oncol 40: 711-720, 2012; DOI: 10.3892/ijo.2011.1270

Following the publication of the above article, a concerned reader drew to the Editor's attention that certain of the western blot assay data shown in Fig. 4G on p. 717 were strikingly similar to data that had appeared in a paper published previously in the journal *Molecular Cancer Therapeutics*, which had been written by different authors at different research institutes. Moreover, there appeared to be as many as five sets of duplicated protein bands in Fig. 4B and C, including the appearance of apparently the same protein band in different orientations in Fig. 4C in one case, and certain data shown in Fig. 4B and G had apparently been re-used in these figure parts.

After having performed an independent assessment of these issues in the Editorial Office, these concerns of the reader were found to have been validated; therefore, the Editor of *International Journal of Oncology* has decided that this paper should be retracted from the publication. After having been in contact with the authors, they accepted the decision to retract the paper. The corresponding author, Claudio Festuccia, takes full responsibility for the raised concerns and clarifies that the other co-authors were not directly involved in the preparation of the submitted figures. The Editor apologizes to the readership for any inconvenience caused.

